# *Bordetella pertussis* Infection and Respiratory Viral Co-Detection Among Children in Türkiye: A Three-Year Epidemiological Study

**DOI:** 10.3390/pathogens15101095

**Published:** 2026-10-21

**Authors:** Meltem Ayaş, Aylin Suran, Ravel Deniz Saygı, Neval Yurttutan Uyar, Sesin Kocagöz

**Affiliations:** 1 Department of Medical Laboratory Techniques, Vocational School of Health Services, Acibadem Mehmet Ali Aydinlar University, Istanbul 34752, Türkiye; meltem.ayas@acibadem.edu.tr; 2 Department of Medical Microbiology, School of Medicine, Acibadem Mehmet Ali Aydinlar University, Istanbul 34752, Türkiye; 3 School of Medicine, Acibadem Mehmet Ali Aydinlar University, Istanbul 34752, Türkiye; aylin.suran@live.acibadem.edu.tr (A.S.); ravel.saygi@live.acibadem.edu.tr (R.D.S.); 4 Acıbadem Labmed Medical Laboratories, Istanbul 34752, Türkiye; 5 Department of Infectious Diseases and Clinical Microbiology, School of Medicine, Acibadem Mehmet Ali Aydinlar University, Istanbul 34752, Türkiye

**Keywords:** *Bordetella pertussis*, whooping cough, pediatric, co-detection, multiplex PCR, epidemiology, Türkiye

## Abstract

Pertussis can be difficult to distinguish from other respiratory infections in children. Describing respiratory viruses detected alongside *Bordetella pertussis* may inform the interpretation of multiplex test results, although molecular co-detection does not establish that both organisms caused the illness. This retrospective laboratory-based study evaluated *B. pertussis* positivity and respiratory viral co-detections among children aged 0–17 years whose respiratory specimens were submitted for multiplex testing to a central laboratory in Türkiye between January 2023 and September 2025. Testing was requested by the referring clinical teams; no standardized symptom-based inclusion criteria were applied. Among 2718 children tested, 61 were positive for *B. pertussis* (2.24%; 95% CI, 1.75–2.87%). Positivity was highest among infants aged <1 year and decreased with increasing age. Third-quarter peaks were observed in 2023 (17/361; 4.7%) and 2024 (4/90; 4.4%). At least one respiratory virus was co-detected in 30 of the 61 positive children (49.2%); rhinovirus/enterovirus and respiratory syncytial virus were the most frequently identified viruses. These findings support continued pertussis surveillance and consideration of *B. pertussis* when interpreting multiplex respiratory test results. The clinical significance of viral co-detection could not be assessed without patient-level clinical outcome data.

## 1. Introduction

Pertussis (whooping cough) is a highly contagious respiratory infection caused by *Bordetella pertussis*, an exclusively human pathogen transmitted primarily through respiratory droplets [1]. Although individuals of all ages can be infected, the disease burden is greatest among children, particularly young infants [2,3]. Infants are at the highest risk of severe disease and account for most pertussis-related hospitalizations and deaths [4]. Despite widespread vaccination programs, *B. pertussis* continues to circulate globally, highlighting the persistent public health importance of pertussis surveillance [3].

Following an incubation period typically ranging from 7 to 10 days, pertussis classically progresses through catarrhal, paroxysmal, and convalescent stages [1,5,6]. The early catarrhal phase is characterized by nonspecific respiratory symptoms and may be clinically indistinguishable from other respiratory infections [4,6,7]. The subsequent paroxysmal phase is characterized by recurrent coughing episodes that may be accompanied by an inspiratory “whoop”, post-tussive vomiting, cyanosis, or apnea, particularly in infants [6,7,8]. Clinical presentation may also vary according to age and vaccination status; vaccinated children generally experience milder disease, whereas adolescents and adults may present primarily with prolonged cough [7,8,9]. The substantial clinical overlap between pertussis and other respiratory infections can complicate etiological diagnosis and supports the use of molecular methods capable of simultaneously detecting multiple respiratory pathogens.

Recent surveillance data indicate a substantial resurgence of pertussis following the marked decline observed during the COVID-19 pandemic. According to the World Health Organization (WHO), 941,565 pertussis cases were reported globally in 2024 [10]. Consistent with this global increase, 209,674 cases were reported across 29 European Union/European Economic Area (EU/EEA) countries in 2024, corresponding to a notification rate of 54.9 per 100,000 population, more than eight times the rate reported in 2023 [11]. Infants aged <1 year were the most affected group (318.5 per 100,000), followed by adolescents aged 10–14 years (204.2 per 100,000) [11]. In Türkiye, reported cases similarly increased from 512 in 2023 to 1588 in 2024 [10]. The factors underlying the recent resurgence are likely multifactorial and remain debated, with waning vaccine-induced immunity, accumulation of susceptible individuals following reduced circulation during the COVID-19 pandemic, reduced natural immune boosting, cyclical disease patterns, pathogen adaptation, and changes in testing capacity and practices proposed as potential contributors [3,11,12].

Vaccination remains the primary strategy for preventing pertussis and reducing severe disease [12]. In Türkiye, acellular pertussis-containing vaccines are administered at 2, 4, and 6 months, followed by booster doses at 18 and 48 months [13,14]. In April 2025, the hexavalent DTaP–IPV–Hib–HepB vaccine replaced the previously used pentavalent formulation for the infant doses [15]. Maternal Tdap vaccination was also introduced into the national programme in April 2025, with one dose recommended at 18–24 weeks of each pregnancy [16]. The booster at 48 months is scheduled before school entry; it should not be interpreted as a requirement for school admission. Although national vaccination coverage has been high, individual vaccination histories and province-specific coverage were unavailable for the present study population. Recent data from Türkiye have documented continued *B. pertussis* activity [2]; however, multicenter laboratory-based data covering the recent resurgence period remain limited.

According to WHO/UNICEF estimates, national coverage with the third dose of a diphtheria–tetanus–pertussis-containing vaccine (DTP3) in Türkiye was 99% in 2019, decreased to 95% in 2021, and returned to 99% in 2023 [17]. In 2023, Türkiye was in the ≥95% DTP3 coverage category, whereas neighboring Greece and Bulgaria were in the 90–94% category and Georgia was in the 80–89% category [18]. These national estimates provide regional context but do not indicate the vaccination status of children in the present study or explain its geographic differences in *B. pertussis* positivity.

National vaccination coverage has been high; however, national estimates cannot establish the vaccination status of children tested in the present hospital network, and differences in coverage across the participating provinces could not be assessed from the present dataset.

Respiratory viral co-detections represent an additional consideration in the diagnosis and epidemiology of pediatric pertussis. Previous studies have reported considerable variation in their frequency, ranging from relatively uncommon detection to viral co-detection in a substantial proportion of pediatric pertussis cases [19,20,21]. Rhinovirus has frequently been identified among the most common viral co-pathogens, although respiratory syncytial virus, parainfluenza viruses, and other respiratory viruses have also been detected [20,21]. The clinical significance of these co-detections remains uncertain; some studies have found no clear association with overall disease severity, whereas others have suggested associations with specific manifestations such as radiologically confirmed pneumonia [20,21]. Contemporary data describing the spectrum of respiratory viral co-detections among pediatric pertussis cases during the recent resurgence in Türkiye remain limited.

Against this background, a three-year laboratory-based epidemiological study was conducted among children tested for respiratory pathogens across a hospital network serving nine provinces of Türkiye. *B. pertussis* positivity, its demographic, temporal, and geographic distribution, and the frequency and spectrum of respiratory viral co-detections were evaluated.

## 2. Materials and Methods

### 2.1. Study Design and Population

This retrospective, laboratory-based study included 2718 children aged 0–17 years whose respiratory specimens were submitted to Acıbadem Labmed Medical Laboratories for multiplex respiratory testing between January 2023 and September 2025. The central laboratory received specimens from hospitals in nine provinces of Türkiye. Children in this age range with an available multiplex test result were included; patients aged ≥18 years were excluded from the pediatric analysis. Testing was requested by the referring clinical teams, without standardized symptom-based inclusion criteria applied by the central laboratory. Laboratory records were checked for repeat testing, and no child was represented more than once.

Demographic and laboratory data, including age, sex, date of testing, testing location, *B. pertussis* PCR results, and results for other respiratory pathogens detected by the multiplex panel, were retrospectively obtained from the laboratory information system. As a central laboratory, Acıbadem Labmed received specimens from multiple hospitals. Detailed clinical records were maintained in the referring hospitals’ information systems and were not accessible for this analysis. Age was calculated at the time of testing. For temporal analyses, samples were grouped by calendar year and quarter (Q1: January–March; Q2: April–June; Q3: July–September; and Q4: October–December). Geographic analyses were based on the province in which the patient was tested.

### 2.2. Molecular Detection of Respiratory Pathogens

All respiratory specimens were recorded as nasopharyngeal swabs in the laboratory information system. Specimens were collected at the referring hospitals as part of routine clinical practice and submitted to the central laboratory for molecular testing. Detailed information regarding the exact anatomical sampling procedure was unavailable in the central laboratory records.

Respiratory specimens were tested using the QIAstat-Dx Respiratory SARS-CoV-2 Panel on the QIAstat-Dx Analyzer 1.0 platform (QIAGEN, Hilden, Germany). The panel included the following respiratory pathogens and subtypes: adenovirus; coronavirus 229E, HKU1, NL63, and OC43; SARS-CoV-2; human metapneumovirus A + B; influenza A, influenza A H1, influenza A H3, influenza A H1N1/pdm09, and influenza B; parainfluenza viruses 1–4; rhinovirus/enterovirus; respiratory syncytial virus A + B; *Bordetella pertussis*; *Chlamydophila pneumoniae*; and *Mycoplasma pneumoniae*. Rhinovirus and enterovirus were reported as a combined rhinovirus/enterovirus target because the assay does not differentiate between them; therefore, their individual frequencies could not be determined. Sample processing, nucleic acid extraction, amplification, detection, and interpretation of results were performed according to the manufacturer’s instructions. A *B. pertussis*-positive result was defined as detection of the corresponding target according to the manufacturer’s interpretation criteria. The exact panel version could not be verified from the available retrospective laboratory records.

### 2.3. Definition of Respiratory Viral Co-Detection

Respiratory viral co-detection was defined as the simultaneous detection of *B. pertussis* and at least one respiratory virus included in the multiplex panel in the same respiratory specimen. When more than one viral target was detected together with *B. pertussis*, all detected viruses were recorded. The term “co-detection” was used rather than “co-infection” because molecular detection of multiple pathogens in the same specimen does not necessarily establish that each detected pathogen was causing active infection.

### 2.4. Statistical Analysis

*B. pertussis* positivity was calculated as the proportion of positive patients among those tested, with 95% confidence intervals (CIs) estimated using the Wilson score method. Age distributions of *B. pertussis*-positive and -negative pediatric patients were compared using the Mann–Whitney U test. Differences in positivity according to sex were evaluated using Fisher’s exact test because of the small number of positive cases.

Temporal variation in *B. pertussis* positivity was evaluated by calendar quarter. Differences among quarters within each year and comparisons of the same quarter across years were assessed using the Fisher–Freeman–Halton exact test for contingency tables with more than two categories and small expected cell counts. For comparisons involving two groups, including the Q4 comparison for the two available years, Fisher’s exact test was used. All tests were two-sided, and a *p* value < 0.05 was considered statistically significant. Statistical analyses were performed using Python version 3.14.3 with NumPy version 2.4.3, and figures were generated using Matplotlib version 3.10.8.

### 2.5. Ethical Approval

The study was conducted in accordance with the Declaration of Helsinki and was approved by the Acıbadem Mehmet Ali Aydınlar University Medical Research Evaluation Board (ATADEK) on 21 May 2026 (Decision No. 2026-09/380).

## 3. Results

### 3.1. Demographic and Geographic Characteristics of Bordetella pertussis Positivity

Among the 2718 children included in the study, *B. pertussis* was detected in 61 (2.24%; 95% CI, 1.75–2.87%). Demographic and geographic characteristics according to *B. pertussis* positivity are summarized in Table 1, and the age distribution of positive cases is shown in Figure 1. *B. pertussis*-positive patients were significantly younger than those who tested negative (median age, 0 vs. 2 years; Mann–Whitney U test, *p* < 0.001), whereas positivity did not differ significantly by sex (Fisher’s exact test, *p* = 1.000). Among the 61 *B. pertussis*-positive children, 54 (88.5%) were infants aged <1 year. More detailed stratification of infants by age in months was not possible because age was available in the laboratory database only in completed years. Positive cases were predominantly observed among infants aged <1 year (Figure 1). Province-specific positivity varied across the hospital network, with the highest positivity observed in Kocaeli (Table 1). Vaccination status was unavailable for all 61 *B. pertussis*-positive children.

### 3.2. Temporal Distribution

Quarterly *B. pertussis* positivity varied over the study period (Figure 2; Table 2). In 2023, positivity differed significantly across quarters (Fisher–Freeman–Halton exact test, *p* < 0.001), increasing from 0% in Q1 to 4.7% in Q3 and subsequently decreasing to 2.6% in Q4. In 2024, positivity was 4.1% in Q1 and reached 4.4% in Q3; however, variation across quarters did not reach statistical significance (*p* = 0.051). In 2025, positivity was 0.6% in Q1, with no positive cases detected in Q2 or Q3; within-year variation was not statistically significant (*p* = 1.000).

Comparisons of corresponding quarters across years showed a statistically significant difference for Q1 (*p* < 0.001), whereas no significant differences were observed for Q2 (*p* = 0.585), Q3 (*p* = 0.364), or Q4 (*p* = 0.088). Data for Q4 were available only for 2023 and 2024 because the study period ended in September 2025.

### 3.3. Respiratory Viral Co-Detections

At least one respiratory viral co-detection was identified in 30 of the 61 *B. pertussis*-positive patients (49.2%). Among these 30 patients, 26 (86.7%) had a single viral co-detection and four (13.3%) had multiple viral co-detections.

Among patients with viral co-detection (*n* = 30), rhinovirus/enterovirus was the most frequently identified single viral co-pathogen (*n* = 13; 43.3%), followed by respiratory syncytial virus (RSV) (*n* = 4; 13.3%) and coronavirus HKU1 (*n* = 2; 6.7%). Coronavirus NL63, coronavirus 229E, human metapneumovirus, human parainfluenza virus 3, human parainfluenza virus 4, influenza B virus, and SARS-CoV-2 were each identified as a single viral co-detection in one patient (*n* = 1; 3.3% each).

Multiple viral co-detections occurred in four patients. Three patients had double viral co-detections: rhinovirus/enterovirus with coronavirus HKU1, rhinovirus/enterovirus with coronavirus OC43, and rhinovirus/enterovirus with human parainfluenza virus 3 (*n* = 1 each). One patient had a triple viral co-detection comprising rhinovirus/enterovirus, influenza A (H3), and influenza A (H1N1) pdm09. The complete profile of respiratory viral co-detections is presented in Table 3.

## 4. Discussion

In this three-year laboratory-based study, *Bordetella pertussis* was detected in 61/2718 (2.24%) children presenting with respiratory symptoms across a hospital network in Türkiye. Three findings were particularly noteworthy: *B. pertussis* positivity was concentrated among infants, detection varied substantially over time and geographically, and 30/61 (49.2%) *B. pertussis*-positive children had at least one respiratory virus detected in the same specimen. Together, these findings provide contemporary evidence that pertussis continues to circulate among symptomatic children in Türkiye and illustrate the complex epidemiological pattern observed during the recent post-pandemic resurgence.

The predominance of pertussis among the youngest children was one of the clearest findings of the present study. Children with *B. pertussis* were significantly younger than those who tested negative, and the highest positivity was observed among infants aged <1 year. This age distribution is clinically important because young infants represent the population most vulnerable to severe pertussis, particularly before completion of the primary vaccination series [4,11]. Recent post-pandemic studies have similarly identified young age and incomplete or absent vaccination as major determinants of severe pediatric pertussis [22]. However, changes in age distribution have also been reported during the recent resurgence. Liu et al., for example, observed increasing proportions of cases among preschool- and school-aged children during 2022–2024 [23], indicating that contemporary pertussis epidemiology may differ considerably between populations and settings.

Notably, 54 of the 61 *B. pertussis*-positive children (88.5%) were infants aged <1 year. This finding should be interpreted in the context of the national immunization schedule in Türkiye, in which pertussis-containing vaccine doses are administered at 2, 4, and 6 months of age, followed by booster doses at 18 and 48 months [13,14]. Thus, infants within the first year of life may differ substantially in the number of vaccine doses for which they are age-eligible. However, age was recorded in completed years in the laboratory dataset, and individual vaccination histories were unavailable. Consequently, infants could not be further stratified by age in months. The proportions of infants who were too young to have received the first dose, were partially vaccinated, or were age-eligible to have completed the primary series could not be determined. This limitation precludes conclusions regarding the relationship between vaccination status and *B. pertussis* positivity in the present cohort.

The finding of predominantly infant disease should therefore not be interpreted as evidence that pertussis circulation is restricted to this age group. Rather, it may reflect the population presenting for multiplex respiratory testing within the hospital network, together with the greater likelihood of testing young symptomatic infants. Nevertheless, the concentration of positivity in infancy reinforces the importance of timely childhood vaccination and strategies that provide protection during the first months of life, including maternal immunization.

A second important observation was the marked temporal variability in *B. pertussis* detection. Positivity peaked during the third quarters of both 2023 and 2024, whereas lower positivity was observed during the first three quarters of 2025. This pattern broadly coincides with the post-pandemic resurgence of pertussis reported in Türkiye and other countries [10,11,12], but the findings also illustrate why short-term temporal patterns should be interpreted cautiously. Recent studies suggest that post-pandemic pertussis circulation does not necessarily follow historical seasonal patterns. Liu et al. reported progressively prolonged periods of increased detection between 2022 and 2024, eventually extending across most of the year, while other pediatric data have demonstrated shifts from previously reported seasonal peaks [23,24].

Several interacting factors may contribute to these altered patterns. Reduced pathogen circulation during COVID-19-related non-pharmaceutical interventions may have increased the pool of susceptible individuals, while subsequent restoration of social contact, changes in healthcare-seeking behavior, increased clinical awareness, and wider use of molecular respiratory testing may all have influenced case detection [23]. Importantly, these mechanisms cannot be distinguished using the laboratory dataset. Furthermore, although third-quarter peaks were observed in both 2023 and 2024, statistically significant quarterly variation was demonstrated only in 2023. Therefore, the findings do not establish a reproducible seasonal pattern. The apparent decline in 2025 should likewise not be interpreted as evidence that the resurgence had ended, because observations for that year were available only through September.

The temporal findings can be viewed alongside population-based surveillance data. Before the study period, the EU/EEA pertussis notification rate was 0.7 cases per 100,000 population in 2022; it rose to 6.7 in 2023 and 54.9 in 2024 [10]. Türkiye reported 512 cases in 2023 and 1588 in 2024 [11]. In neighboring Bulgaria, the notification rate increased from 0.3 per 100,000 in 2023 to 42.2 in 2024, while Greece reported an increase from 0.1 to 4.2 per 100,000 over the same years [10]. These surveillance trends provide regional context for the study period. However, the positivity observed among children selected for hospital-based testing cannot be compared directly with population notification rates or used to infer cross-border transmission.

Geographic heterogeneity was also apparent. Although Istanbul accounted for 1392/2718 (51.2%) children, the highest number of positive cases and the highest positivity rate were observed in Kocaeli (33/569; 5.80%). This finding is notable but should not be interpreted as evidence of a higher population incidence of pertussis in Kocaeli. The study was based on patients tested within a private hospital network rather than population-based surveillance, and geographic differences may therefore reflect referral patterns, local testing practices, differences in healthcare utilization, or localized transmission. Nevertheless, the observation demonstrates that pertussis activity within the same healthcare network was not geographically uniform and provides a rationale for more detailed regional surveillance using population-based denominators.

One of the main findings of the present study was the high frequency of respiratory viral co-detection. At least one respiratory virus was identified in 30/61 (49.2%) of *B. pertussis*-positive children, most frequently rhinovirus/enterovirus, followed by RSV. Previous studies have reported widely varying frequencies and spectra of respiratory pathogens detected together with *B. pertussis*. Frassanito et al. reported viral co-detection in 47.2% infants hospitalized with pertussis, remarkably similar to the finding, whereas Jiang et al. detected respiratory viruses in 40.1% affected children, with rhinovirus being the predominant virus [20,21]. More recent pediatric surveillance has likewise identified human rhinovirus among the principal pathogens detected together with *B. pertussis* [23].

The frequency of respiratory viral co-detection observed in the present study (30/61; 49.2%) is broadly comparable with findings from several pediatric studies, although direct comparisons should be interpreted in the context of differences in age, clinical setting, sampling strategy, and diagnostic methods. In Italy, Frassanito et al. reported respiratory viral co-detection in 25 of 53 (47.2%) hospitalized infants younger than 180 days with *B. pertussis* infection. Nasopharyngeal washings were tested for 14 respiratory viruses by real-time RT-PCR, with rhinovirus being the most frequently identified viral pathogen [20]. Similarly, a prospective study conducted in southeastern China during 2017–2019 enrolled children younger than 24 months hospitalized with lower respiratory tract infection and identified at least one respiratory virus in 81 of 202 (40.1%) *B. pertussis*-positive children; rhinovirus was again the predominant viral co-detection (25.7%) [21]. More recent data from southern China during 2023–2024 also demonstrate substantial co-detection of respiratory pathogens among pediatric pertussis cases, with the distribution varying by age and hospitalization status; rhinovirus was the most frequently detected respiratory virus overall, whereas RSV was relatively more frequent among younger hospitalized children [25]. These findings are relevant to the present cohort, in which most *B. pertussis*-positive cases occurred during infancy and rhinovirus/enterovirus represented the most frequent viral co-detection. Nevertheless, differences in study populations and testing strategies are important: the present study included children aged 0–17 years tested using nasopharyngeal swabs and a multiplex molecular respiratory panel, whereas several previous studies focused specifically on hospitalized infants or young children with lower respiratory tract infection. Thus, variation in reported co-detection frequencies likely reflects not only geographical and temporal epidemiological differences but also differences in age distribution, clinical setting, inclusion criteria, specimen type, and the range of respiratory pathogens included in molecular testing.

The similarity in the predominance of rhinovirus across several studies is interesting, although it should not be interpreted as evidence of a specific biological interaction. Studies examining individual pathogen associations have produced heterogeneous results. Muloiwa et al., for example, found that children with *B. pertussis* had a greater number of respiratory organisms detected overall, but only *Chlamydia pneumoniae*, *Mycoplasma pneumoniae*, and parainfluenza viruses were independently associated with *B. pertussis* detection; no significant positive association with RSV was demonstrated [26]. Thus, the high proportion of viral detections in the present cohort may reflect the broader polymicrobial nature of pediatric respiratory illness rather than a pathogen-specific interaction.

This distinction is particularly important when interpreting multiplex molecular assays. Detection of nucleic acid from two pathogens in the same respiratory specimen does not establish that both are simultaneously responsible for the clinical illness. Viral shedding may persist after a preceding infection, and molecular assays may identify organisms of uncertain clinical relevance. Similar limitations have been emphasized in previous multiplex-PCR studies of pediatric pertussis [24,26]. The term “co-detection” was therefore considered more appropriate than “co-infection” for the present dataset.

The analytical performance of multiplex respiratory panels should also be considered when interpreting these findings. Multicenter evaluations of the QIAstat-Dx Respiratory Panel using nasopharyngeal swab specimens have demonstrated high overall diagnostic performance, including high agreement for *B. pertussis* [27]. However, analytical sensitivity may vary among respiratory targets, and comparative studies have shown that some pathogens, particularly those present at low loads, may be less consistently detected by multiplex assays [28]. Lower agreement has also been reported for multiple-pathogen detections compared with single-pathogen detections [28]. Therefore, although all specimens in the present study were tested using the same platform and specimen type, some low-abundance viral targets may have remained undetected. Consequently, the observed frequency of respiratory viral co-detection may represent an underestimate of the true frequency.

At the same time, the frequent identification of additional respiratory viruses has practical diagnostic implications. Early pertussis symptoms overlap considerably with those of common viral respiratory infections, and detection of a respiratory virus should therefore not automatically exclude *B. pertussis* as an additional diagnosis. Conversely, identification of *B. pertussis* does not necessarily explain the entire clinical presentation when another pathogen is simultaneously detected. Multiplex syndromic testing may be particularly useful in this setting because it permits simultaneous evaluation of clinically overlapping respiratory pathogens [24]. Recent pediatric studies have similarly emphasized the potential value of multiplex testing in the setting of changing post-pandemic epidemiology. However, because clinical severity and outcomes were not available in the present study, the effect of viral co-detection on disease severity or clinical course could not be determined.

The present study has several strengths. It captures a recent period encompassing the post-pandemic resurgence of pertussis, applies the same multiplex molecular platform across a hospital network, and includes children tested in nine provinces. This enabled demographic, temporal, geographic, and viral co-detection patterns to be examined within a single dataset. The inclusion of all tested symptomatic children, rather than only *B. pertussis*-positive cases, also allowed positivity rates to be interpreted relative to the population undergoing respiratory testing.

Several limitations should be considered when interpreting these findings. First, this retrospective, laboratory-based study was conducted within a private hospital network, and the tested population was not a population-based sample of children in Türkiye. Referral patterns, healthcare utilization, and testing practices may have influenced overall and province-specific positivity. Second, individual vaccination records and detailed clinical data were maintained in the referring hospitals’ information systems and were not accessible to the central laboratory for this analysis. Thus, vaccination status could not be classified, and symptoms, disease severity, clinical course, hospitalization, respiratory support, NICU/PICU admission, and outcomes at discharge could not be evaluated. Third, multiplex PCR detects pathogen nucleic acids but cannot establish whether each detected organism contributed to the current illness; prolonged viral shedding and incidental detection cannot be excluded. Consequently, this study cannot determine whether viral co-detection was associated with greater pertussis severity, much less establish a causal relationship. Finally, geographic comparisons used the number of patients tested within the hospital network rather than population denominators, and data for 2025 were available only through September.

Prospective multicenter surveillance integrating molecular testing with individual vaccination history, maternal immunization status, clinical severity, and outcomes would help determine which children remain most vulnerable during contemporary pertussis resurgence. Population-based surveillance is also needed to establish whether the temporal and geographic patterns observed in the hospital network reflect broader regional transmission. Finally, longitudinal studies combining pathogen detection with clinical follow-up may help distinguish clinically meaningful co-infection from transient or incidental molecular co-detection.

## 5. Conclusions

*Bordetella pertussis* continued to circulate among children tested for respiratory symptoms in Türkiye during 2023–2025, with the highest positivity observed among infants aged <1 year. Temporal and geographic variation was observed during the study period, although these patterns should be interpreted within the context of a hospital-based population and incomplete data for 2025. The frequent co-detection of respiratory viruses, particularly rhinovirus/enterovirus and RSV, highlights the complexity of pediatric respiratory infections and the potential utility of multiplex molecular testing. These findings provide contemporary epidemiological data from Türkiye and reinforce the importance of continued pertussis surveillance and timely childhood vaccination. Further population-based studies incorporating individual vaccination status and clinical outcomes are needed to clarify regional transmission patterns and the clinical significance of respiratory viral co-detections.

## Figures and Tables

**Figure 1:**
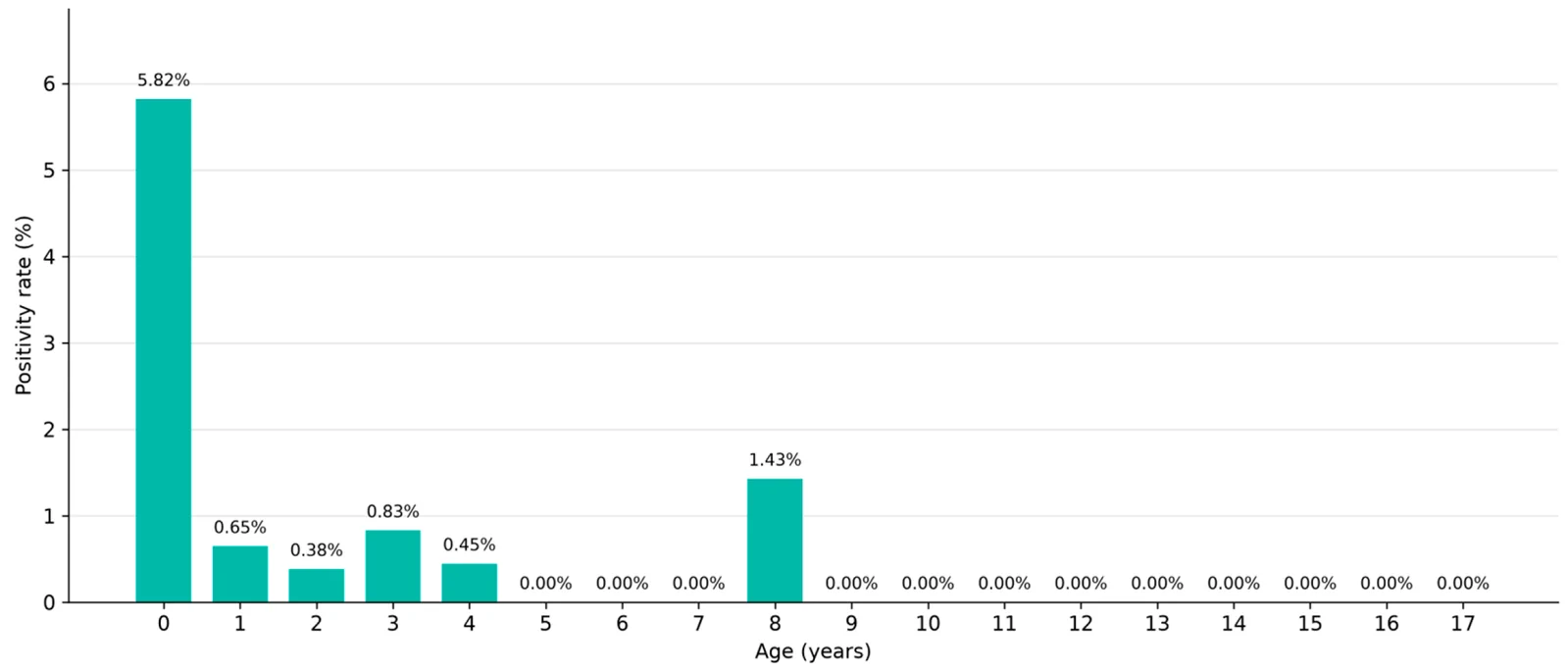
Age-group distribution of children with *Bordetella pertussis*-positive results (*n* = 61). Percentages represent the proportion of the 61 positive cases within each age group and do not represent age-specific positivity rates.

**Figure 2:**
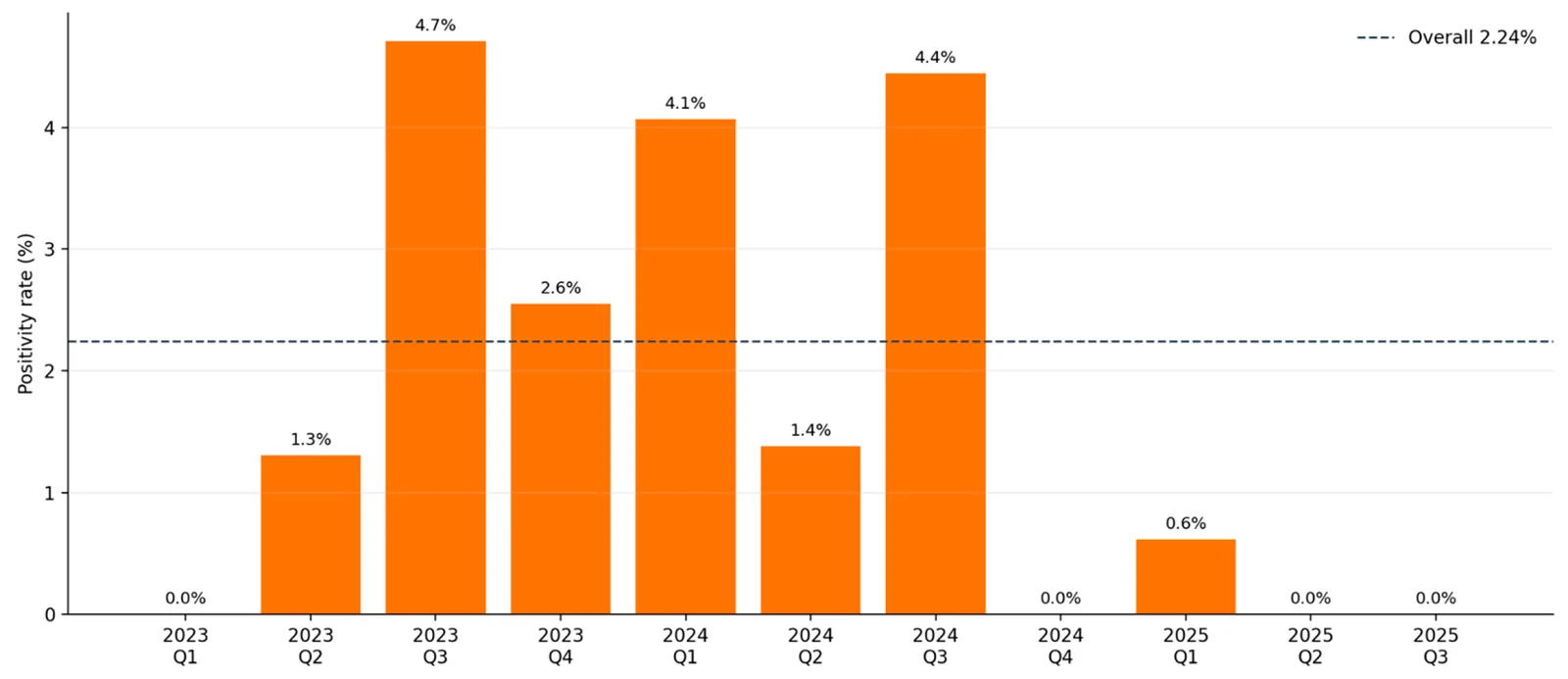
Quarterly *Bordetella pertussis* positivity, 2023–September 2025. Q1, January–March; Q2, April–June; Q3, July–September; Q4, October–December. The dashed line indicates overall positivity (61/2718; 2.24%). No Q4 data were available for 2025 because the study period ended in September.

**Table 1:** *Bordetella pertussis* positivity among pediatric patients (0–17 years) by subgroup, Türkiye, 2023–2025.

Characteristic	Tested (*n*)	Positive (*n*)	Positivity % (95% CI)
Overall	2718	61	2.24 (1.75–2.87)
Sex			
Female	1187	27	2.27 (1.57–3.29)
Male	1531	34	2.22 (1.59–3.09)
Province (top three)			
Kocaeli	569	33	5.80 (4.16–8.03)
Istanbul	1392	21	1.51 (0.99–2.30)
Bursa	374	4	1.07 (0.42–2.72)

**Table 2:** Quarterly *Bordetella pertussis* positivity among children, January 2023–September 2025.

Period	Positive (*n*)	Tested (*n*)	Positivity (%)
2023 Q1	0	277	0.00
2023 Q2	5	383	1.31
2023 Q3	17	361	4.71
2023 Q4	16	627	2.55
2024 Q1	16	393	4.07
2024 Q2	2	145	1.38
2024 Q3	4	90	4.44
2024 Q4	0	127	0.00
2025 Q1	1	163	0.61
2025 Q2	0	110	0.00
2025 Q3	0	42	0.00
Overall	61	2718	2.24

Positivity = positive/tested × 100; no Q4 data were available for 2025.

**Table 3:** Respiratory viral co-detections among the 61 patients positive for *Bordetella pertussis* (30 patients with ≥1 co-detection).

Co-Detection	*n*	% of Co-Detected (*n* = 30)
Single viral co-detection	26	86.7
Rhinovirus/enterovirus	13	43.3
Respiratory syncytial virus (RSV)	4	13.3
Coronavirus HKU1	2	6.7
Coronavirus NL63	1	3.3
Coronavirus 229E	1	3.3
Human metapneumovirus	1	3.3
Human parainfluenza virus 3	1	3.3
Human parainfluenza virus 4	1	3.3
Influenza B virus	1	3.3
SARS-CoV-2	1	3.3
Double viral co-detection	3	10.0
Rhinovirus/enterovirus + coronavirus HKU1	1	3.3
Rhinovirus/enterovirus + coronavirus OC43	1	3.3
Rhinovirus/enterovirus + human parainfluenza virus 3	1	3.3
Triple viral co-detection	1	3.3
Rhinovirus/enterovirus + influenza A (H3) + influenza A (H1N1)pdm09	1	3.3
Total with ≥1 viral co-detection	30	100

Percentages are calculated among the 30 patients with at least one viral co-detection (49.2% of all 61 *B. pertussis*-positive patients).

## Data Availability

The data supporting the findings of this study are not publicly available due to patient privacy and ethical restrictions. De-identified data may be made available from the corresponding author upon reasonable request, subject to institutional and ethical approval.

## References

[1] World Health Organization Pertussis. https://www.who.int/health-topics/pertussis.

[2] Yakut S., Ulaba A., Yıldız Zeyrek F. (2025). Hastanemizdeki boğmaca olgularının değerlendirilmesi: Beş yıllık analiz. Mikrobiyoloji Bül..

[3] Wang S., Zhang S., Liu J. (2025). Resurgence of pertussis: Epidemiological trends, contributing factors, challenges, and recommendations for vaccination and surveillance. Hum. Vaccines Immunother..

[4] Decker M.D., Edwards K.M. (2021). Pertussis (whooping cough). J. Infect. Dis..

[5] Marcdante K.J., Kliegman R.M., Schuh A.M. (2022). Nelson Essentials of Pediatrics.

[6] Vijayan V., Daley S.F. (2026). Pertussis. StatPearls.

[7] Otar G., Kılıç A., Yıldız İ., Varkal M.A., Devecioğlu E. (2014). Boğmaca enfeksiyonunun tanı ve tedavisi. Çocuk Derg..

[8] Cherry J.D., Heininger U., Feigin R.D., Cherry J.D., Demmler-Harrison G.J., Kaplan S.L. (2014). Pertussis and other *Bordetella* infections. Textbook of Pediatric Infectious Diseases.

[9] Yürük Atasoy P., Artuk C., Sönmez C., Nar Ötgün S., Turan M., Kılıç S. (2024). Yetişkinlerde uzun süren öksürüğün *Bordetella pertussis* enfeksiyonu ile ilişkisi: Kültür, PCR ve serolojik yöntemler ile kesitsel bir değerlendirme. Türk Mikrobiyoloji Cemiy. Derg..

[10] World Health Organization Pertussis—Reported Cases and Incidence. WHO Immunization Data Portal. https://immunizationdata.who.int/.

[11] European Centre for Disease Prevention and Control (2026). Pertussis. Annual Epidemiological Report for 2024.

[12] Duda-Madej A., Łabaz J., Topola E., Bazan H., Viscardi S. (2025). Pertussis—A re-emerging threat despite immunization: An analysis of vaccine effectiveness and antibiotic resistance. Int. J. Mol. Sci..

[13] Republic of Türkiye, Ministry of Health The Expanded Programme on Immunization (EPI). https://covid19asi.saglik.gov.tr/EN-80230/the-expanded-programme-on-immunization-epi.html.

[14] Republic of Türkiye, Ministry of Health Aşı Takvimi. https://asi.saglik.gov.tr/bagisiklama-programi-ve-asi-takvimi/asi-takvimi.html.

[15] Republic of Türkiye, Ministry of Health Altı Hastalığa Karşı Tek Aşı Uygulaması [Implementation of a Single Vaccine Against Six Diseases]. https://adanaism.saglik.gov.tr/TR-345596/alti-hastaliga-karsi-tek-asi-uygulamasi.html.

[16] Republic of Türkiye, Ministry of Health Gebelikte Tetanoz Difteri Boğmaca Aşısı [Tetanus, Diphtheria and Pertussis Vaccination During Pregnancy]. https://adanaism.saglik.gov.tr/TR-345597/gebelikte-tetanoz-difteri-bogmaca-asisi.html.

[17] World Health Organization, United Nations Children’s Fund WHO and UNICEF Estimates of National Immunization Coverage: Türkiye, 2023. https://cdn.who.int/media/docs/default-source/country-profiles/immunization/2024-country-profiles/immunization-2024-tur.pdf.

[18] United Nations Children’s Fund (2024). In Focus: Immunization.

[19] Heininger U., Burckhardt M.A. (2011). *Bordetella pertussis* and concomitant viral respiratory tract infections are rare in children with cough illness. Pediatr. Infect. Dis. J..

[20] Frassanito A., Nenna R., Nicolai A., Pierangeli A., Tozzi A.E., Stefanelli P., Carsetti R., Concato C., Schiavoni I., Midulla F. (2017). Infants hospitalized for *Bordetella pertussis* infection commonly have respiratory viral coinfections. BMC Infect. Dis..

[21] Jiang W., Wu M., Chen S., Li A., Wang K., Wang Y., Chen Z., Hao C., Shao X., Xu J. (2021). Virus coinfection is a predictor of radiologically confirmed pneumonia in children with *Bordetella pertussis* infection. Infect. Dis. Ther..

[22] Gatto A., Buonsenso D., Rulli E., Prokopchuk M., Zampino G., Sanguinetti M., Sali M., La Sorda M. (2025). Factors associated with more severe disease in infants and children with pertussis in the post-pandemic era: A single-center retrospective study. Pathogens.

[23] Liu M., Wei X., Sun J., Liu Y., Cheng L., Han Y., Ma X. (2025). Molecular epidemiological characteristics of pertussis in a pediatric hospital in Jinan City, China, 2022 to 2024. Front. Public Health.

[24] Xue M., Wei X., Wang B., Liu M., Zhang Y., Ma X. (2026). Clinical characteristics analysis of pertussis and *Mycoplasma pneumoniae* infection in children. Front. Cell. Infect. Microbiol..

[25] Lai W., Guo J., Zhou X., Luo Y., Zheng M., Mai Q., Xiong J., Luo M. (2025). Co-infection in pediatric pertussis during 2023 and 2024 in southern China: Pathogen distribution characteristic and influence on manifestation of pertussis. Front. Microbiol..

[26] Muloiwa R., Dube F.S., Nicol M.P., Hussey G.D., Zar H.J. (2020). Co-detection of *Bordetella pertussis* and other respiratory organisms in children hospitalised with lower respiratory tract infection. Sci. Rep..

[27] Leber A.L., Lisby J.G., Hansen G., Relich R.F., Schneider U.V., Granato P., Young S., Pareja J., Hannet I. (2020). Multicenter evaluation of the QIAstat-Dx Respiratory Panel for detection of viruses and bacteria in nasopharyngeal swab specimens. J. Clin. Microbiol..

[28] Gosert R., Koller R., Meyer J., Dräger S., Ramette A., Bingisser R., Nickel C.H., Bassetti S., Tschudin Sutter S., Keller P.M. (2024). Multicenter evaluation of the QIAstat-Dx and the BioFire multiplex panel tests for the detection of respiratory pathogens. J. Med. Virol..

